# Neighborhood characteristics and the risk of psoriasis: A systematic review

**DOI:** 10.1016/j.jdin.2023.08.011

**Published:** 2023-08-25

**Authors:** Owen Dan Luo, Zainab Ridha, Abdulhadi Jfri, Mohsen Rezaeian, Anastasiya Muntyanu, Julien Ringuet, Elena Netchiporouk

**Affiliations:** a Department of Medicine, McGill University, Montreal, Quebec, Canada; b Division of Dermatology, McGill University Health Centre, Montreal, Quebec, Canada; c Division of Dermatology, King Saud Bin Abdulaziz University for Health Sciences, Riyadh, Saudi Arabia; d Social Medicine Department, Rafsanjan University of Medical Sciences, Rafsanjan, Iran; e Department of Experimental Medicine, McGill University, Montreal, Quebec, Canada; f Centre de Recherche Dermatologique du Québec Métropolitain, Québec, Quebec, Canada

**Keywords:** air pollution, built environment, deprivation, incidence, psoriasis, severity, urbanization

*To the Editor:* Psoriasis affects 1% to 5% of the North American population.^1^ The association between lifestyle (eg, physical activity, diet, alcohol, and smoking) and psoriasis/its comorbidities is well established.^2^^,^^3^ However, it is increasingly recognized that behavioral risk factors arise in a larger context of socioeconomic, cultural, and environmental determinants of health.^4^ Research in chronic diseases (eg, diabetes mellitus, metabolic syndrome) highlighted the importance of the living environment (LE) (ie, physical and socioeconomic conditions in which people live, work, and play) as a critical element to address population-level health differences. LE contributes to health inequity (ie, unjust and potentially avoidable differences in health outcomes among different populations). We aimed to conduct a systematic review to understand the impact of LE on psoriasis.

MEDLINE, EMBASE, Web of Science, and CINAHL databases were searched on September 20, 2022, by ODL and ZR for studies exploring the association between LE and the prevalence, incidence, or severity of psoriasis. Preferred Reporting Items for Systematic Reviews and Meta-Analyses (PRISMA) guidelines were followed. Search strategy is detailed in Supplementary Tables I-III, available via Mendeley at https://doi.org/10.17632/vk94dhftw5.1. Studies’ quality was appraised using the Quality Assessment Tool for Quantitative Studies.^5^

Of 8 studies included (Fig 1 and Supplementary Table IV, available via Mendeley at https://doi.org/10.17632/vk94dhftw5.1 summarize the PRISMA flow diagram and studies’ details, respectively), 2 ascertained the association between urban versus rural residence and psoriasis risk with conflicting results. Two articles investigated the association between neighborhood socioeconomic conditions and psoriasis. People residing in high- and medium-deprivation neighborhoods (ie, deprivation of essential resources and/or goods) were more likely to have psoriasis whereas patients from the lowest income quartiles had a more severe disease.

**Fig 1:**
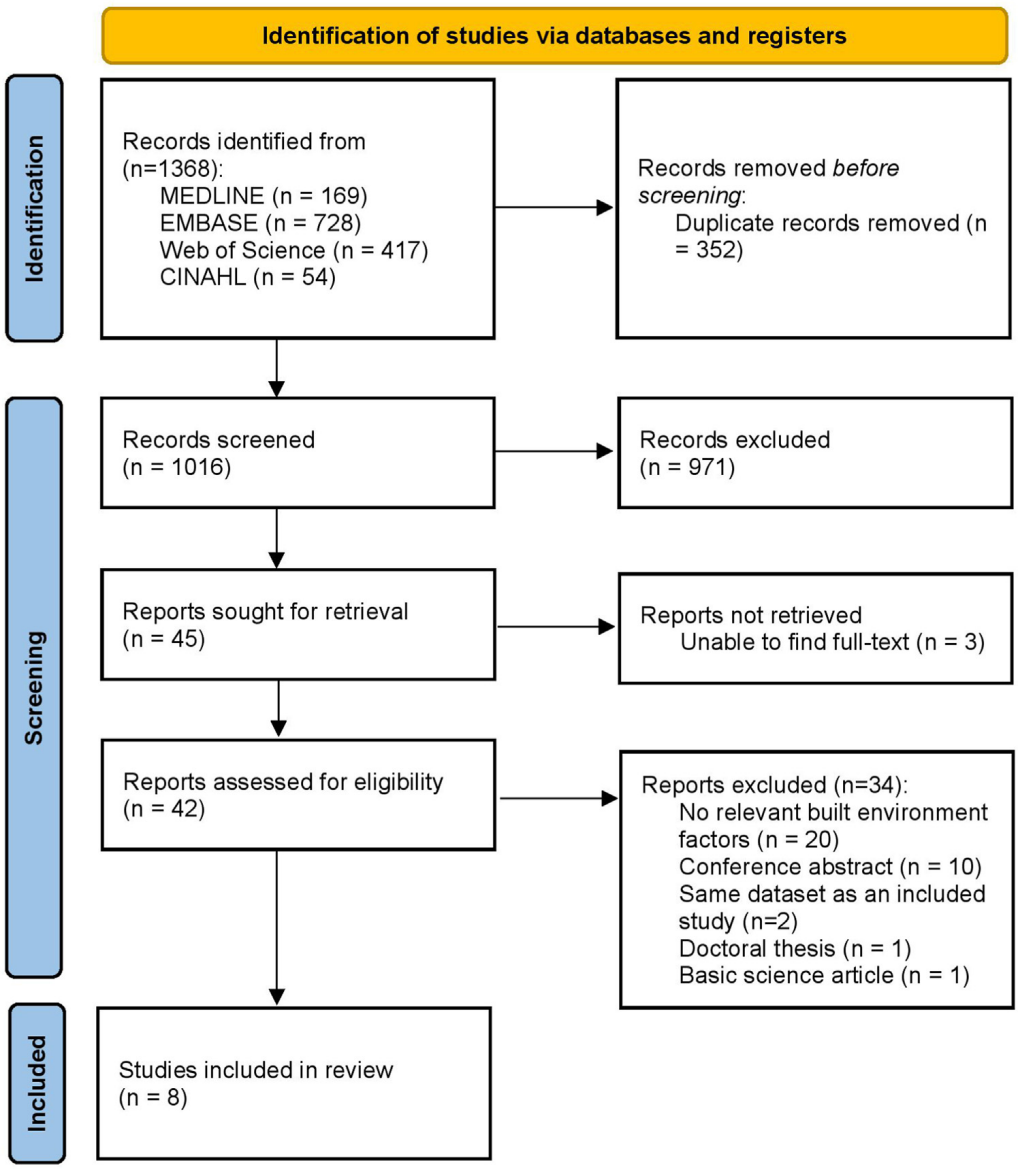
Preferred Reporting Items for Systematic Reviews and Meta-Analyses (PRISMA) flow diagram.

Four studies researched the association between air quality and psoriasis exacerbations. Particulate matter (PM_2.5_ and PM_10_) and NO_2_ were associated with a modest increase in outpatient visits and hospital admissions in South Korea and China. Italian studies demonstrated higher concentrations of all air pollutants (eg, PM_2.5_, PM_10_, CO, NO_2_, other nitrogen oxides, benzene) prior to psoriasis flares versus regular outpatient visits as well as daily increases of 10 μg/m^3^ in air pollutants were associated with therapeutic decisions such as dose increments or treatment changes.

The available evidence suggests that neighborhoods with socioeconomic deprivation may be associated with a higher psoriasis risk and severity, whereas communities with worse air quality may increase the risk of psoriasis flare. However, this data should be interpreted with caution due to limited number of studies on the topic and at least moderate risk of bias identified across studies included (Supplementary Table V, available via Mendeley at https://doi.org/10.17632/vk94dhftw5.1) owing to data source, study design, patients’ number, and/or statistical analyses. Despite psoriasis disproportionately affecting North American, Western European, and Australasian populations, we identified no studies from these regions. Studying LE characteristics such as environmental (eg, air/noise/light pollution, greenness), built environment-related (eg, man-made buildings and spaces), and socioeconomic neighborhood characteristics (eg, material, social instability, and deprivation) as determinants of psoriasis incidence/severity is important to advance our understanding of population-level determinants of psoriatic disease spectrum. This is essential to reduce health disparities in chronic skin disease such as psoriasis and reduce the individual, societal and economic burden of this common and morbid disease.

## Conflicts of interest

None disclosed.
